# High levels of depressive symptoms among people with lower limb lymphoedema in Rwanda: a cross-sectional study

**DOI:** 10.1093/trstmh/traa139

**Published:** 2020-11-21

**Authors:** Maya Semrau, Gail Davey, Ursin Bayisenge, Kebede Deribe

**Affiliations:** Centre for Global Health Research, Brighton & Sussex Medical School, University of Sussex, Falmer, Brighton, BN1 9PS, UK; Centre for Global Health Research, Brighton & Sussex Medical School, University of Sussex, Falmer, Brighton, BN1 9PS, UK; School of Public Health, College of Health Sciences, Addis Ababa University, P.O. Box 9086, Ethiopia; Centre for One Health, University of Global Health Equity, Kigali Heights, Plot 772 KG 7 Avenue, 5th Floor, PO Box 6955, Kigali, Rwanda; Centre for Global Health Research, Brighton & Sussex Medical School, University of Sussex, Falmer, Brighton, BN1 9PS, UK; School of Public Health, College of Health Sciences, Addis Ababa University, P.O. Box 9086, Ethiopia

**Keywords:** depression, lymphoedema, mental health, morbidity, prevalence, Rwanda

## Abstract

**Background:**

There is a growing body of evidence that mental distress and disorder are common among people with lower limb lymphoedema, although no research has been conducted on this subject in Rwanda.

**Methods:**

This research was embedded within a mapping study to determine the national prevalence and geographical distribution of podoconiosis in Rwanda. Using a cluster sampling design, adult members of households within 80 randomly selected sectors in all 30 districts of Rwanda were first screened and 1143 patients were diagnosed with either podoconiosis (n=914) or lower limb lymphoedema of another cause (n=229). These 1143 participants completed the Patient Health Questionnaire (PHQ)-9 to establish the prevalence of depressive symptoms.

**Results:**

Overall, 68.5% of participants reported depressive symptoms- 34.3% had mild depressive symptoms, 24.2% had moderate, 8.8% moderately severe and 1.2% severe depressive symptoms. The mean PHQ-9 score was 7.39 (SD=5.29) out of a possible 0 (no depression) to 27 (severe depression). Linear regression showed unemployment to be a consistently strong predictor of depressive symptoms; the other predictors were region (province), type of lymphoedema and, for those with podoconiosis, female gender, marital status and disease stage.

**Conclusions:**

Levels of depressive symptoms were very high among people with lower limb lymphoedema in Rwanda, which should be addressed through holistic morbidity management and disability prevention services that integrate mental health, psychosocial and economic interventions alongside physical care.

## Introduction

Podoconiosis is a neglected tropical disease (NTD) that is common among barefoot subsistence farmers who have long-term exposure to irritant red clay tropical soils^1^ in areas where ancient volcanic deposits have weathered at high altitude^2^ and where there is heavy rainfall. Podoconiosis results in lower limb lymphoedema, i.e. swelling of the lower leg and feet, as do other NTDs such as lymphatic filariasis (LF) and leprosy, which—unlike podoconiosis—are caused by parasites and *Bacilli*, respectively.

Rwanda is one of the countries where podoconiosis has only recently been mapped, with an estimated overall prevalence of 68.5 per 100 000 people or 0.069%, which amounts to around 6429 people with podoconiosis, spread widely across Rwanda.^3^ Other countries in which podoconiosis is widespread include Ethiopia, Cameroon, Kenya, Tanzania and Uganda,^4^ with approximately 4 million cases worldwide across at least 32 countries.^4,5^ Globally, lymphoedema caused by either LF or leprosy occurs in around 15 million^6^ and 200 000^7^ people, respectively.

In addition to the physical disability associated with lower limb lymphoedema, there is a large mental health and psychosocial burden for patients as well as their families and communities, which can include mental distress,^8^ depression,^9^ stigma^10–14^ and loss of economic productivity.^15–17^ For example, a recent study in Cameroon by our group found that 38.6% of people with podoconiosis or lymphoedema of another cause displayed at least mild depressive symptoms.^18^ Other previous research reported that 12.6% of podoconiosis patients in northern Ethiopia had depressive symptoms and that 5.2% were at risk of suicide, compared with only 0.7% and 0.4% of their healthy neighbours, respectively.^9^ In Nigeria, 20% of people with LF have been reported to have depression compared with 3.1–5.2% in the general adult population.^19^ Mental distress has also been found to be significantly higher among people with podoconiosis in Ethiopia compared with healthy controls.^8^ What is more, a global study on the burden of depression associated with LF estimated that the psychological and emotional effects of LF were double those of the physical health burden linked to the disease.^20^ This comorbidity with mental disorder and overall reduced mental well-being, together with the associated stigma that is commonly experienced by people with lower limb lymphoedema, can act as a major barrier to accessing and adhering to morbidity management and disability prevention (MMDP) services.^21,22^ However, research on this subject is still in its infancy and—to our knowledge—no study on the mental health implications of podoconiosis or lower limb lymphoedema of another cause has to date been conducted in Rwanda.

This study aims to address this gap by reporting on the prevalence of depressive symptoms among people with lower limb lymphoedema in Rwanda, as well as providing comparisons between people affected by podoconiosis and those with lower limb lymphoedema of another cause.

## Materials and Methods

### Design

This research was part of a larger population-based countrywide cross-sectional survey that aimed to determine the national prevalence and geographical distribution of podoconiosis in Rwanda.^3^ The larger mapping study used a cluster sampling design, whereby at least two sectors (proportional to the number of sectors per district to adjust for sector size) were randomly selected from within each of the 30 districts of Rwanda. The research reported here was embedded within the larger mapping study and aimed to estimate the prevalence of depressive symptoms among those people who were diagnosed with either podoconiosis or lower limb lymphoedema of another cause during the larger study.

### Setting

The study was conducted in 80 randomly selected sectors (out of a possible 416) within all 30 districts of Rwanda. The screening stage took place within participants’ households, while the second verification/diagnosis stage was conducted in a private room at the nearest public health centre.

### Sample

For the larger mapping study, all adults within randomly selected sectors aged >15 y who had lived in the area for at least 10 y (to omit those individuals who might have acquired the leg swelling elsewhere) were invited to participate, excluding those who were not able to respond, for example, due to terminal illness or a severe mental disorder. The research reported here involved 1143 participants who were identified as having either podoconiosis or lower limb lymphoedema of another cause in the larger study.

### Procedure

As part of the larger mapping study, during the first screening stage, all households in the randomly selected sectors were visited in June or July 2017 by trained community health workers who documented residence, gender, age and whether leg swelling of any type was present or not, with people who showed swelling of one or both lower limbs being recorded as suspected podoconiosis cases. In total, 1360 612 individuals were screened, of whom 640 882 were men (47.1%) and 719 730 were women (52.9%). Of these, 1143 (0.08%) were identified to be possible podoconiosis cases, i.e. individuals with lower limb lymphoedema.

In the second stage, during November and December 2017, the 1143 suspected cases were verified as either podoconiosis (n=914; 80.0%) or lower limb lymphoedema of another cause (n=229; 20.0%) by locally recruited expert clinical diagnostic teams, each of which included four health workers, a medical doctor, a nurse, a laboratory technician and a team leader, using procedures that had previously been employed during a mapping study in Cameroon.^18,23^ All individuals with suspected podoconiosis were physically examined, and a diagnosis of podoconiosis or lymphoedema of another cause was established through this physical examination, as well as via history and disease-specific tests. A podoconiosis case was defined as a person residing in the surveyed district for at least 10 y who had bilateral, asymmetrical lymphoedema of the lower limb lasting for >1 y, negative Filariasis Test Strip (Alere; Scarborough, ME, USA) and Wb123 tests, and a history of any of the signs and symptoms associated with podoconiosis (for more details on this, including information on the aetiologies of those with lymphoedema of another cause, see the larger mapping study conducted by Deribe et al.^3^). For disease staging of podoconiosis, the staging system of Tekola et al. (2008)^24^ was used, where 1 denotes the least severe and 5 the most severe disease stage. In addition, participants’ sociodemographic data and information on disease characteristics were collected via questionnaires, including individual-level data on age, gender, education, occupation, place of residence, shoe-wearing and foot hygiene practices, age at onset of swelling, family member(s) (living or dead) with a history of leg swelling, the type of swelling (ascending upwards from the foot, or descending down the leg from the upper leg or groin area), self-reported chronic illness such as heart disease, kidney disease or diabetes, clinical diagnoses of known causes of lymphoedema (such as congenital disorders, leprosy and postoperative lymphoedema) and the cooccurrence of swelling in other parts of the body such as the hands, face and scrotum (hydrocele), household data on water, sanitation and hygiene, as well as geographical coordinates from surveyed communities via smartphones.

Furthermore, all 1143 participants identified as having lower limb lymphoedema were administered the Patient Health Questionnaire (PHQ)-9 as a measure of depressive symptoms. The PHQ-9 has been used, tested and validated as a measure of depression in a wide range of global settings.^25,26^ The scale covers nine questions, which ask respondents how often they have been affected by a set of nine problems over the last 2 wk, for example, having little interest or pleasure in doing things, feeling tired or having little energy, having a poor appetite or overeating and having trouble concentrating. There is also an additional question (which is not part of the overall scoring) that asks the respondent how difficult any depression symptoms made it for them to complete various activities of daily life. The PHQ-9 scores were classified in this study according to categories that have been widely used,^9^ as follows: no depression (0–4), mild (5–9), moderate (10–14), moderately severe (15–19) and severe depression (20–27). It is these PHQ-9 results that this paper reports on (see Deribe et al.^3^ for the findings of the larger mapping study).

All study materials were translated into and administered in the Kinyarwanda language. Data were collected using the LINKS software package (version 1.4.2; Secure Data Kit, Atlanta, GA, USA) installed onto Android smartphones. This required data collectors to complete all compulsory questions before being able to move on to the next one.

Ethical approval was obtained as part of the larger mapping study from the Rwanda National Ethics Committee and the Brighton & Sussex Medical School Research Governance and Ethics Committee, Brighton, UK. All participants either gave their written informed consent to take part in the study or, where participants were illiterate, they confirmed their consent via a thumbprint and a signature from a literate witness. Where participants were aged <18 y, they gave their consent verbally and a parent or guardian provided written consent.

### Analysis

Data analyses were conducted using IBM SPSS 25 and 26 (IBM Corp; Armonk, New York, USA). A significance level of 0.05 was employed for all analyses. Descriptive analyses (frequencies and percentages for categorical variables and means with standard deviations for continuous variables) were used to present the sociodemographic and disease characteristics of participants. χ^2^ (categorical variables; using Fisher's exact test where the expected cell counts were <10) and independent t-tests (continuous variables) were employed to assess whether there were any differences in sociodemographic and disease characteristics and PHQ-9 scores between participants who had podoconiosis vs those who had lymphoedema of another cause.

To determine the prevalence of depressive symptoms, the mean total PHQ-9 score was calculated. Cronbach's alpha was computed as a measure of the internal consistency of the PHQ-9 to assess whether the PHQ-9 was able to reliably measure depressive symptoms within this population; a higher Cronbach's alpha score indicates a higher likelihood that all items in the scale measure the same underlying construct.

Standard multivariate linear regression was employed, to assess the association between participants’ sociodemographic and disease characteristics and depressive symptoms. Two multivariate regression models were run; in the first, all 1143 participants were included, and in the second only those participants diagnosed with podoconiosis (n=914) were included so that disease stage could be added into the model (as these data were not available for participants with lymphoedema of another cause). PHQ-9 score (depressive symptoms) was the dependent variable in both models, and the following factors were included as independent variables: age (continuous variable), gender (male/female), geographical region (five provinces), marital status (married/single/divorced/widowed), employment status (employed/unemployed), literacy (literate/illiterate), type of lymphoedema (podoconiosis/lymphoedema of another cause) (first model only) and stage of disease (stage 1/stage 2/stage 3 and above) (second model only). No variable selection was performed and independent variables included in the models were chosen based on prior literature and expert knowledge. Where any independent variables had categories with <5% of participants, these were regrouped so that all categories contained at least 5% of participants. Dummy variables were created in SPSS for those categorical variables with >2 levels before they were entered into the regression models. Assumptions of both regression models were checked, including homoscedasticity, multicollinearity, independence and normality of residuals; outliers were also examined.

## Results

Table 1 shows the sociodemographic and disease characteristics of the 1143 people identified as having either podoconiosis (n=914; 80.0%) or lower limb lymphoedema of another cause (n=229; 20.0%). The only two of the sociodemographic or disease characteristics listed in Table 1 for which there was a statistically significant difference between those who had podoconiosis compared with those who had lymphoedema of another cause were employment (χ^2^=12.12, d.f.=1; p < 0.0001) and occupation (χ^2^=17.42, d.f.=8; p=0.026). Those with podoconiosis were more likely not to be working (11.1%) than those with lymphoedema of another cause (3.5%). In accordance with this, for those occupation categories related to being in work (farmer and ‘employed other’), there were less people with podoconiosis proportionally to those with lymphoedema of another cause (Table 1).

**Table 1.** Sociodemographic and disease characteristics of people with lower limb lymphoedema in Rwanda

	Total	Podoconiosis	Lymphoedema
Characteristics	(n=1143)	(n=914)	of another cause (n=229)
Gender			
Male	359 (31.4%)	276 (30.2%)	83 (36.2%)
Female	784 (68.6%)	638 (69.8%)	146 (63.8%)
Age, mean in years	53.41 (SD=17.56)	53.03 (SD=17.47)	54.93 (SD=17.88)
Region (province)			
Kigali	35 (3.1%)	26 (2.8%)	9 (3.9%)
Northern	245 (21.4%)	195 (21.3%)	50 (21.8%)
Eastern	281 (24.6%)	223 (24.4%)	58 (25.3%)
Southern	282 (24.7%)	239 (26.1%)	43 (18.8%)
Western	300 (26.2%)	231 (25.3%)	69 (30.1%)
Marital status			
Married	546 (47.8%)	432 (47.3%)	114 (49.8%)
Widowed	323 (28.3%)	257 (28.1%)	66 (28.8%)
Single	185 (16.2%)	155 (17.0%)	30 (13.1%)
Divorced	89 (7.8%)	70 (7.7%)	19 (8.3%)
*Religion*			
*Christian*	*1123 (98.3%)*	*898 (98.2%)*	*225 (98.3%)*
*Muslim*	*10 (0.9%)*	*7 (0.8%)*	*3 (1.3%)*
*Other*	*10 (0.9%)*	*9 (1.0%)*	*1 (0.4%)*
*Level of education*			
*No formal education*	*797 (69.7%)*	*642 (70.2%)*	*155 (67.7%)*
*Primary school*	*319 (27.9%)*	*249 (27.2%)*	*70 (30.6%)*
*Secondary school*	*20 (1.7%)*	*19 (2.1%)*	*1 (0.4%)*
*Tertiary education*	*7 (0.6%)*	*4 (0.4%)*	*3 (1.3%)*
Literate			
Yes	379 (33.2%)	293 (32.1%)	86 (37.6%)
No	764 (66.8%)	621 (67.9%)	143 (62.4%)
Employment			
Yes	1034 (90.5%)	813 (88.9%)	221 (96.5%)
No*	109 (9.5%)	101 (11.1%)	8 (3.5%)
*Occupation*			
*Farmer*	*1007 (88.1%)*	*793 (86.8%)*	*214 (93.4%)*
*Employed other (non-farmer)*	*27 (2.4%)*	*20 (2.2%)*	*7 (3.1%)*
*Student*	*11 (1.0%)*	*11 (1.2%)*	*0*
*Jobless*	*91 (8.0%)*	*84 (9.2%)*	*7 (3.1%)*
*Retired*	*7 (0.6%)*	*6 (0.7%)*	*1 (0.4%)*
*Mean number of years in current location*	*47.82 (SD=19.13)*	*47.59 (SD=19.21)*	*48.76 (SD=18.82)*
*Family member with history of leg swelling*			
*Yes*	*481 (42.1%)*	*390 (42.7%)*	*91 (39.7%)*
*No*	*662 (57.9%)*	*524 (57.3%)*	*138 (60.3%)*
Disease stage**			
Stage 1	N/A	274 (30.0%)	N/A
Stage 2		443 (48.5%)	
Stages 3–5		197 (21.6%)	

* This includes students and retired participants.

** The staging system by Tekola et al. (2008)^24^ was used, where 1 denotes the least severe disease stage and 5 marks the most severe disease stage.

Variables in italics were not included in the regression models but are included here for informational purposes.

In regard to depressive symptoms, the mean PHQ-9 score among all 1143 participants was 7.39 (SD=5.29), ranging between 0 and 24 (out of a possible maximum score of 27, which denotes very severe depressive symptoms). For the additional question on the PHQ-9, which asks how difficult any symptoms of depression made it for the respondent to carry out their work, take care of things at home, or get along with other people, the mean score was 1.68 (SD=0.77), on a scale of 0 (not difficult at all), 1 (somewhat difficult), 2 (very difficult) and 3 (extremely difficult). Cronbach's alpha for the PHQ-9 was 0.8, which is considered to demonstrate good internal consistency.

Using a PHQ-9 score of ≥5 as cut-off, 783 people (68.5%) with lower limb lymphoedema (n=1143) were classified as having at least mild depressive symptoms. Using a PHQ-9 score of ≥10 as a screening indicator for major depressive disorder, 391 people (34.2%) were classified as being in this category, with 115 (10.0%) having either moderately severe or severe depressive symptoms (Table 2). Among people who scored above the screening cut-off score of 10, the mean PHQ-9 score was 13.34 (SD=2.99).

**Table 2.** Depressive symptoms reported by people with lower limb lymphoedema in Rwanda

Depressive symptoms	Total	Podoconiosis	Lymphoedema
(based on PHQ-9 scores)	(n=1143)	(n=914)	of another cause (n=229)
Mean (SD)	7.39 (5.29)	7.25 (5.13)	7.95 (5.86)
No depression (0–4)	360 (31.5%)	293 (32.1%)	67 (29.3%)
Mild (5–9)	392 (34.3%)	316 (34.6%)	76 (33.2%)
Moderate (10–14)	276 (24.1%)	225 (24.6%)	51 (22.3%)
Moderately severe (15–19)	101 (8.8%)	72 (7.9%)	29 (12.7%)
Severe (20–27)	14 (1.2%)	8 (0.9%)	6 (2.6%)

Both multivariate standard linear regression models were accepted, since all assumption checks were satisfactory, although both models were only able to account for a small amount of the variance in depressive symptoms (Tables 3 and 4). The only variables that proved to be significant predictors for depressive symptoms in both of the models that were run were (1) unemployment, which strongly predicted depressive symptoms in both models (Tables 3 and 4), i.e. participants who were not in work had significantly higher PHQ-9 scores on average than those who were in work (when including all 1143 participants, the mean PHQ-9 score for people not in work was 9.02 [SD=5.63] compared with a mean of 7.22 [SD=5.23] for people who were working), and (2) region—specifically, participants in Western Province reported significantly more depressive symptoms than those in Eastern Province (mean=7.88, SD=5.02 vs mean=6.77, SD=5.35, respectively, when including all 1143 participants; for reference, the mean PHQ-9 scores for the other three provinces were: Kigali [mean=7.69, SD=5.60], Northern [mean=7.30, SD=5.81] and Southern [mean=7.54, SD=5.0], none of which were significantly different from Eastern Province).

**Table 3.** Multiple regression analysis for all participants with lower limb lymphoedema in Rwanda (n=1143)

PHQ-9	B	95% CI for B	SE B	ß	R^2^	Adj-R^2^
Model					0.04	0.03***
Constant	4.92***	2.99 to 6.84	0.98			
Age	0.02	−0.002 to 0.04	0.01	0.07		
Gender (male: reference group/female)	0.36	−0.37 to 1.08	0.37	0.03		
Region (reference group: Eastern)						
Kigali	0.94	−0.91 to 2.78	0.94	0.03		
Northern	0.43	−0.48 to 1.33	0.46	0.03		
Southern	0.71	−0.16 to 1.58	0.44	0.06		
Western	1.15**	0.30 to 2.01	0.44	0.10**		
Marital status (reference group: married)						
Single	0.62	−0.37 to 1.61	0.50	0.04		
Divorced	0.77	−0.43 to 1.97	0.61	0.04		
Widowed	0.64	−0.19 to 1.48	0.42	0.06		
Employed (yes: reference group/no)	1.73***	0.67 to 2.80	0.54	0.10***		
Literate (yes: reference group/no)	0.61	−0.06 to 1.28	0.34	0.05		
Podoconiosis (yes/no: reference group)	−0.84*	−1.61 to −0.08	0.39	−0.06*		

Model=‘Enter’ method in SPSS; B=unstandardized regression coefficient; SE B=standard error of the coefficient; ß=standardized coefficient; R^2^*=*coefficient of determination; Adj-R^2^*=*adjusted R^2^. *p<0.05; **p<0.01; ***p<0.001.

**Table 4.** Multiple regression analysis for participants with podoconiosis in Rwanda (n=914)

PHQ-9	B	95% CI for B	SE B	ß	R^2^	Adj-R^2^
Model					0.06	0.05***
Constant	4.13***	2.08 to 6.17	1.04			
Age	0.02	−0.01 to 0.04	0.01	0.05		
Gender (male: reference group/female)	0.82*	0.05 to 1.62	0.41	0.07*		
Region (reference group: Eastern)						
Kigali	1.37	−0.69 to 3.42	1.05	0.04		
Northern	0.36	−0.62 to 1.34	0.05	0.03		
Southern	0.90	−0.02 to 1.82	0.47	0.08		
Western	1.26**	0.32 to 2.19	0.48	0.11**		
Marital status (reference group: married)						
Single	0.71	−0.34 to 1.75	0.53	0.05		
Divorced	1.78**	0.47 to 3.08	0.67	0.09**		
Widowed	0.94*	0.04 to 1.84	0.46	0.08*		
Employed (yes: reference group/no)	1.75***	0.67 to 2.82	0.55	0.11***		
Literate (yes: reference group/no)	0.54	−0.19 to 1.28	0.37	0.05		
Disease stage (reference group: stage 3 and above)						
Stage 1	−0.78	−1.72 to 0.17	0.48	−0.07		
Stage 2	−1.31**	−2.18 to −0.45	0.44	−0.13**		

Model=‘Enter’ method in SPSS; B=unstandardized regression coefficient; SE B=standard error of the coefficient; ß=standardized coefficient; R^2^*=*coefficient of determination; Adj-R^2^*=*adjusted R^2^. *p<0.05; **p<0.01; ***p<0.001.

In addition, in the regression model that was run with all participants (n=1143), the type of lymphoedema significantly predicted depressive symptoms, whereby those with lymphoedema of another cause had higher levels of depressive symptoms on average than those with podoconiosis (Tables 2 and 3).

In the model that was run with people affected by podoconiosis only (n=914) (Table 4), additional variables that were predictive of depressive symptoms (as well as unemployment and region) were: female gender (women had mean PHQ-9 scores of 7.58 [SD=5.19] compared with mean PHQ-9 scores of 6.51 [SD=4.93] among men); marital status—specifically, participants who were divorced (mean PHQ-9=8.54, [SD=4.12]) and widowed (mean PHQ-9=8.16 [SD=5.21]) had higher levels of depressive symptoms on average that those who were married (mean PHQ-9=6.49 [SD=5.09]) (for reference, the mean PHQ-9 score for people who were single was 7.30 [SD=5.2], but this was not significantly different compared with being married); and disease stage, i.e. those with stage 3 or higher podoconiosis had significantly higher levels of depressive symptoms on average (mean PHQ-9=8.03 [SD=5.59]) than those with stage 2 podoconiosis (mean PHQ-9=6.96 [SD=5.17]) (for reference, people with stage 1 podoconiosis had a mean PHQ-9 score of 7.17 [SD=4.67], but this score was not significantly different compared with stage 3 or higher).

## Discussion

Our study found very high levels of depressive symptoms among people with lower limb lymphoedema in Rwanda, both for podoconiosis and lymphoedema of another cause. Over two-thirds of participants reported at least mild depressive symptoms. A quarter showed moderate depressive symptoms and every 10th participant displayed moderately severe or severe depressive symptoms, indicating that around a third of participants fit the screening classification for major depressive disorder. In terms of how this affected functioning, on average, participants considered depressive symptoms to make their daily activities somewhat to very difficult.

These figures are higher than those that some prior studies in other countries have reported for people with lower limb lymphoedema. For instance, our previous study in Cameroon, which employed a very similar design to the research reported in this paper, found that 38.6% of people with lower limb lymphoedema displayed at least mild depressive symptoms.^18^ Studies in northern Ethiopia with podoconiosis patients^9^ and in Nigeria with LF patients^19^ reported rates of 12.6% and 20% for depression, respectively. One partial explanation for the higher figures in our Rwandan study may be that rates of depressive symptoms within the general population, even in those without chronic disease, might be higher in Rwanda than elsewhere because a large part of the population who survived the 1994 genocide are still experiencing mental disorders such as depression, panic disorder and post-traumatic stress disorder^27^; indeed, a recent nationwide mental health survey in Rwanda estimated prevalence rates for depression to be 11.9% in the general population and 35% among genocide survivors,^27^ while the aforementioned studies from Ethiopia^9^ and Nigeria^19^ reported depression rates of 0.7% and 3.1–5.2% among healthy neighbours and the general population, respectively. Another factor may be that some of the other studies already mentioned used more rigorous methods to establish depression rather than simply reporting depressive symptoms as in our study; for example, in the Ethiopian study, the PHQ-9 was administered at two time points 2 wk apart and depression was only deemed to be present if indicated at both time points, resulting in more conservative estimates.^9^ In line with this, a recent study by our group in northern Ethiopia that used the PHQ-9 at one time point found even higher rates of depressive symptoms (of 86.5%) among people with podoconiosis, LF and leprosy^28^ compared with the Rwandan study reported here. Nevertheless, given that levels of depressive symptoms in the Rwandan study appear to be far higher than those for the general population in Rwanda, this suggests that people with lower limb lymphoedema in Rwanda face a larger mental health burden.

We found that unemployment was a consistently strong predictor for depressive symptoms among people with lower limb lymphoedema in Rwanda. This is in line with previous research, which has also reported unemployment as being significantly associated with depressive symptoms or depression, including among people with podoconiosis and lower limb lymphoedema of another cause in Cameroon^18^ and among people with LF in Nigeria.^19^ However, as highlighted previously,^18^ causality between unemployment and depressive symptoms for people with lower limb lymphoedema cannot be established based on these studies due to their cross-sectional design. It would be interesting and helpful for this association to be explored further in future research.

Other variables that we found to be predictors for depressive symptoms in the regression models that we ran were region (depressive symptoms were higher among people in Rwanda's Western Province compared with Eastern Province), the type of lymphoedema (participants with lower limb lymphoedema of another cause reported higher levels of depressive symptoms than those with podoconiosis) and, for podoconiosis patients only, female gender, marital status (those who were divorced or widowed had higher levels of depressive symptoms than those who were married) and disease stage (those with stage 3 or higher podoconiosis reported more depressive symptoms). Our findings on female gender and disease stage are consistent with a previous study from Ethiopia, which reported that mental distress was higher among women with podoconiosis compared with men and that mental distress was lowest among those with the middle stages of podoconiosis (although the sample size was not sufficiently large to adequately test this)^29^; the same result was found in our sample. Conversely, another prior study in Ethiopia found that the stage of disease had no impact on depression scores for people affected by podoconiosis.^30^ In regard to region, in keeping with our findings, the recent Rwanda Mental Health Survey reported that depression was higher among genocide survivors from Western Province (along with those from Kigali and Southern Province) compared with those from Eastern Province (along with those from Northern Province)^27^; the reasons for this are not clear and should be explored, along with further investigation of those other factors mentioned above that may contribute to levels of depressive symptoms among people with podoconiosis and lower limb lymphoedema of another cause.

The linear regression models that we employed were only able to account for a very small part of the variance in depressive symptoms. Important predictors for depressive symptoms among this population group in Rwanda were therefore absent from the model. Based on findings from prior research with other populations in Rwanda, possible factors that may be important specifically within the Rwandan context as predictors of depressive symptoms and that were not included within our model could be experiences of past traumatic experiences and grief linked to the genocide against the Tutsi in 1994,^31,32^ poverty and marginalisation^32^ and functional impairment.^33^ Other important factors may include a family history of mental illness,^29^ disability^30^ and experience of stigma.^13,29^ It is plausible—although untested—that these factors may also be predictive of depressive symptoms among people with lower limb lymphoedema in Rwanda. Future research to explore these interactions further among people with lymphoedema would be helpful.

Our study has several limitations, in addition to the cross-sectional design already mentioned and the limited ability of the regression models to explain depressive symptoms in this sample. Another weakness was that we were unable to include a control group as a comparator for depressive symptoms, for example, healthy neighbours, as this was beyond the scope of the larger mapping study within which this research was embedded. A further limitation of our study was the way in which depressive symptoms were measured, which may have led to a bias in the reporting of depressive symptoms, including that the psychometric properties of the PHQ-9 have not been established in this participant group (although the scale has been tested, validated and used in a wide range of populations) and that the methods used to measure depressive symptoms were not as rigorous as in other studies (see above), which may have resulted in an over-reporting of depressive symptoms. Indeed, recent systematic review evidence recommends use of the PHQ-9 for literate populations, but does not support its use so strongly among non-literate populations,^26^ as was the case in our study where only one-third of participants could read and write. Conversely, people with such severe mental disorders that they were not able to respond were excluded from the current study, which could potentially have led to an under-reporting of the most severe levels of depressive symptoms (although the extent and implications of this exclusion criterion are unknown, since the number of participants who were excluded and the reasons for their exclusion were not formally documented). A final limitation is that no mental health staff were included in the implementation of the research since it was conducted as part of the larger podoconiosis mapping study in Rwanda.

### Conclusions

Despite some limitations, our study demonstrates—to our knowledge for the first time—the high levels of depressive symptoms among people in Rwanda with lower limb lymphoedema due to either podoconiosis or another cause. This highlights the importance of considering mental health, psychosocial and economic interventions within MMDP packages of care, ensuring that care for people with lower limb lymphoedema is holistic and addresses not only the physical health consequences of the disease, but also the mental health, psychosocial and economic outcomes.

## Data Availability

The data used in this study were generated through a collaboration between the Rwanda Biomedical Center and Brighton and Sussex Medical School (BSMS). All data sharing requests are reviewed and approved by them. To initiate the data access process, please contact the BSMS Research Governance and Ethics Committee: rgec@bsms. ac.uk, who will provide guidance for accessing the data.
